# Physiological stress reactivity and recovery: Some laboratory results transfer to daily life

**DOI:** 10.3389/fpsyg.2022.943065

**Published:** 2022-08-15

**Authors:** Melanie Bamert, Jennifer Inauen

**Affiliations:** Department of Health Psychology and Behavioral Medicine, Institute of Psychology, University of Bern, Bern, Switzerland

**Keywords:** stress, daily life, heart rate variability, vagal tank theory, stressful events, temporal dynamics, physiological stress response

## Abstract

Stress is a prevalent theme in our daily lives and is related to numerous negative health outcomes. Laboratory research has studied the physiological stress response extensively with objective measures such as vagally-mediated heart rate variability (vmHRV). Recently, the vagal tank theory emerged as a promising approach to predicting adaptive vmHRV levels around stressful events. This study aimed to investigate whether the predictions of the vagal tank theory about vmHRV during stress reactivity and recovery translate into naturalistic stressful events in daily life. Sixty-seven students wore an EcgMove 4 sensor for 4 days to measure vmHRV. Through a combination of device-based and self-report assessment, vmHRV data were segmented into before, during, and after stressful events. VmHRV segments were analyzed with multilevel modeling, accounting for physiological and psychological covariates. VmHRV before stressful events predicted more adaptive vmHRV during the event but not vmHRV recovery afterwards. The results therefore partially support the vagal tank theory's predictions with data from daily life and allow recommendations for future studies of real-world stress reactivity and recovery. The value of intraindividual variations in vmHRV as predictors of adaptive stress response is underscored by these findings and could inform future interventions that seek to increase momentary vmHRV.

## Introduction

Stress is a widespread issue in today's world and has been described as the modern day hidden epidemic (Kalia, 2002). Consistent with this notion, 25% of people working in Europe report being stressed during most or even all their working time (Vargas et al., 2014). These numbers are particularly concerning because stress can have many undesirable effects, such as increased cortisol levels (Wettstein et al., 2020), impairment of the immune system (Dragoş and Tănăsescu, 2010), decreased cognitive functions (e.g., Rantanen et al., 2021), lower mental health (e.g., Karyotaki et al., 2020), and disturbed sleep (Li et al., 2019). To better understand how stress becomes harmful through, for example, repetition or prolongation, one promising approach is to examine repeated short-term stressful events (Rohleder, 2019). Understanding how people react to and recover from stressful events is therefore important. However, whereas stress reactivity and recovery processes are in principle understood in controlled laboratory contexts, it is unknown to what extent this translates into daily life.

Stress is a complex concept. Stress has been defined as individuals' physiological reaction to stimuli (e.g., Selye, 1956), in terms of stimuli that are considered stressful (e.g., daily hassles, Kanner et al., 1981), and also as a transactional process, where stress is understood as a product of cognitive appraisal processes (Lazarus and Folkman, 1984). In this paper, we will focus on short-term events that people cognitively appraise as stressful, and related physiological reactions. When events that people experience as stressful are considered (subsequently simply referred to as stressful events), there are distinct temporal dynamics that can be distinguished: (a) resting before the stressful event, (b) reactivity during the stressful event, (c) recovery after the stressful event (Laborde et al., 2018), and (d) pile-up (i.e., encountering additional stressful events before having recovered from the first one; Smyth et al., 2018). Various aspects of these temporal stress dynamics can be investigated, such as the physiological stress response or mood. In laboratory settings, this has been done with psychophysiological methods that allow the physiological stress response to be measured (e.g., Crowley et al., 2011; Campbell and Ehlert, 2012; Castaldo et al., 2015; Abdelall et al., 2020).

A way of investigating one part of the physiological stress response is by measuring heart rate variability (HRV). HRV reflects the variation in duration of the time intervals between heartbeats (Malik and Camm, 1995). Meta-analyses support the validity of HRV as an indicator of stress. For example, a meta-analysis that included neuroimaging studies showed that HRV relates to brain areas associated with cognitively appraising a situation as stressful (Kim et al., 2018). Further, a systematic review from the field of occupational psychology showed that increased work stress is linked to decreased vmHRV (Järvelin-Pasanen et al., 2018). HRV can be a sensitive indicator of stress because it is influenced by the autonomic nervous system. Stress is associated with a reduction in vagus nerve activity, which can be measured by looking at vagally-mediated HRV (vmHRV) (Thayer and Lane, 2000; Thayer et al., 2009; Smith et al., 2020). Measuring HRV is pain-free, economical, noninvasive and reliable in both laboratory and real-life settings (Castaldo et al., 2015; Laborde et al., 2017; Nakao, 2019). It also provides continuous high-resolution data to investigate the temporal dynamics of stress reactivity and recovery (Ursin and Eriksen, 2004). An unobtrusive measure such as vmHRV could help lay the groundwork for ecological momentary interventions (Heron and Smyth, 2010) and just-in time adaptive interventions (Nahum-Shani et al., 2018) to regulate stress. This is especially promising because consumer-grade devices such as smartwatches measuring vmHRV, and physiological and psychological covariates could play an important role in the future to make interventions based on vmHRV widely accessible.

**Figure 1:**
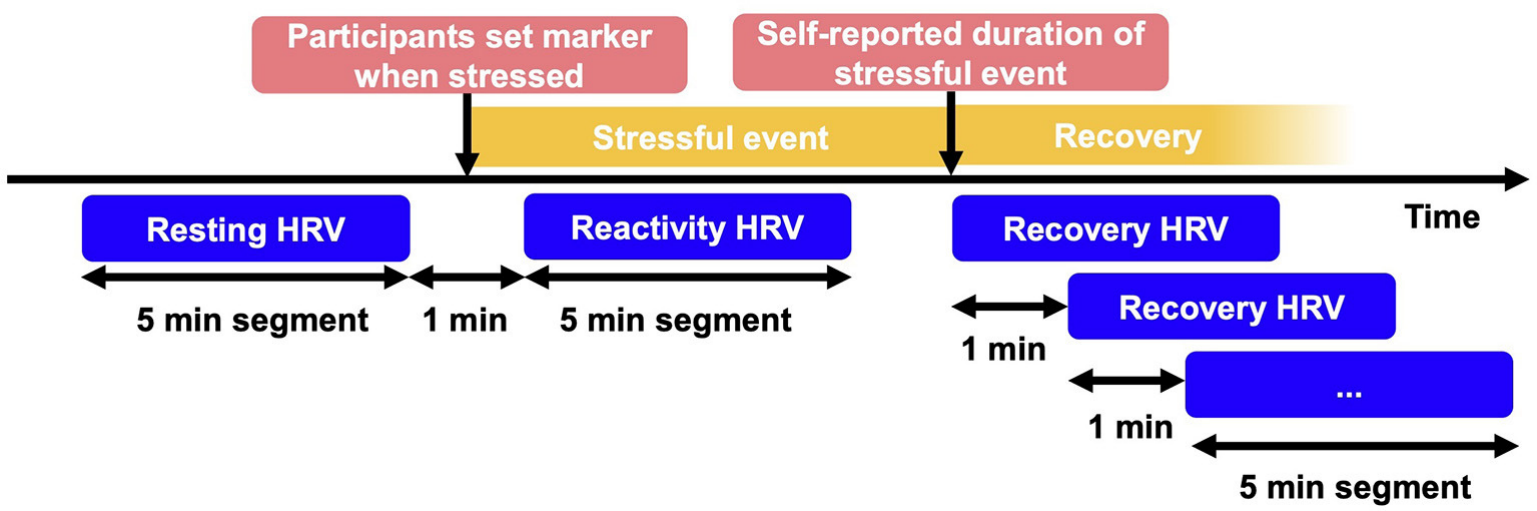
Operationalization of resting, reactivity and recovery HRV. HRV, heart rate variability; min, minutes. The 5-min segments for resting and reactivity HRV were identified through the setting of markers. Start of recovery was identified by additionally considering self-report of event duration. To calculate recovery time, 5-min segments during recovery were calculated until return to resting HRV was reached.

Based on laboratory studies measuring HRV and existing theoretical considerations, theoretical frameworks such as the vagal tank theory (Laborde et al., 2018) have recently emerged. Vagal tank theory makes predictions about adaptive stress reactivity and recovery around stressful events (see Figure 1) according to considerations from social psychology, cognitive psychology, and neurophysiology. Vagal tank theory focuses on vmHRV as one part of the physiological stress response because vagal activity seems crucial in the process of adaptively responding to stressful events based on sensory data (Yuan and Silberstein, 2016; Laborde et al., 2018). Vagal tank theory conceptualizes adaptive stress reactivity and recovery as responses with optimal physiological functioning and resource use (e.g., of executive or metabolic functions), which leads to the effective meeting of the demands of a stressful event.

For resting, vagal tank theory (Laborde et al., 2018) postulates that higher vmHRV values are more adaptive because they have been shown to be linked to better self-regulation, which can be defined as the psychophysiological mechanisms permitting goal-directed behavior in varying situations (e.g., stressful events) (Thayer et al., 2009). These mechanisms include for example executive functioning and coping with stress. Because of this, vagal tank theory also proposes higher vmHRV to be predictive of more adaptive stress reactivity and recovery.

During reactivity, Laborde et al. (2018) underlined how several changes in vmHRV can be adaptive depending on the requirements of the situation. Specifically, according to the theory, the adaptiveness of the patterns vary based on metabolic demands of the situation. During stressful events that require no physical activity but that rely on executive functions (e.g., decision making under pressure), both an increase and a smaller decrease in vmHRV during stress reactivity is assumed to be adaptive. This is expected because a smaller decrease or even increase in vmHRV reflects higher resource availability, and thus better self-regulation. In turn, during stressful events that require physical activity (e.g., engaging in a strenuous workout) a large decrease in vmHRV during stress reactivity is assumed to be adaptive. Thereby, the body can more effectively meet the metabolic demands required under such conditions (Porges, 2007). Overall, these assumptions about adaptive vmHRV underline how a general “higher vmHRV is better” is too simplistic when investigating vmHRV and that an interaction with the demands of the situation needs to be taken into account. Empirical evidence from laboratory studies supports the theory's predictions for vmHRV reactivity (e.g., Laborde et al., 2014; Park et al., 2014; Mosley et al., 2017; Spangler and McGinley, 2020).

Regarding adaptive recovery, the vagal tank theory (Laborde et al., 2018) defined fast restoration of resting levels of vmHRV after a stressful event as adaptive. Fast restoration reflects having sufficient resources to effectively recover and meet potential subsequent stressful events. The exception to this is if vmHRV increased during the stressful event. Vagal tank theory understands an increase in vmHRV during a stressful event as a mechanism of training, resulting in higher vmHRV resting levels. Therefore, remaining above vmHRV resting levels for longer periods after the stressful event is considered to be more adaptive than shorter periods, because it indicates a longer training period, resulting in greater vmHRV resting levels. Again, these assumptions emphasize how a general “faster vmHRV recovery” after a stressful event is oversimplified when investigating vmHRV and that an interaction effect needs to be taken into account. The theory's assumptions for recovery are supported by empirical evidence from laboratory studies (e.g., Stanley et al., 2013; Berna et al., 2014).

Although laboratory research has many advantages, such as allowing continuous supervision of participants and systematic and highly controlled variation of independent variables, doubt remains about the extent to which insights gained in the laboratory can be generalized to stress in everyday life (Wilhelm and Grossman, 2010). First, stress stimuli used in laboratory settings, although diverse, might not be as personally relevant to participants as naturalistic real-life stressful events, thus calling into question the external and ecological validity of the findings. Second, the predictions of theories about stress response based on laboratory research may be too simplistic to extend to daily life. For example, physical activity requirements of stressful events have been conceptualized and operationalized as distinct categories, such as presence of physical activity vs. no physical activity, whereas physical activity in daily life is a continuous phenomenon. Also, vmHRV is influenced by many factors playing an especially large role in daily life contexts such as the consumption of certain substances (alcohol, nicotine, and caffeine), breathing pace, exposure to loud noise, or body posture (Task Force, 1996; Sammito et al., 2014; Laborde et al., 2017). Finally, measuring naturalistic stressful events in daily life makes it possible to observe the pile-up of stressful events, which can interfere with recovery after a stressful event and thus represents an important temporal dynamic of stress in daily life (Smyth et al., 2018). Stress in everyday life has been studied predominantly with insufficient temporal resolution to allow examination of its dynamics. For example, the physiological stress response using vmHRV has been investigated aggregated over extended time windows such as 24-h or daytime vs. nighttime (e.g., Vrijkotte et al., 2000; Orsila et al., 2008; Uusitalo et al., 2011; Hayano et al., 2018). However, this temporal resolution does not allow testing predictions about adaptive vmHRV stress reactivity and recovery related to stressful events in daily life. There have been some recent studies investigating vmHRV dynamics in daily life in a way that covers parts of the assumptions made by vagal tank theory, such as a study by Spangler et al. (2018) looking at vmHRV reactivity during stressful shooting tasks in U.S. Army Soldiers.

In summary, vagal tank theory assumes that resting vmHRV predicts vmHRV reactivity during a stressful event, and vmHRV recovery after the stressful event. These relationships are moderated. The relationship of resting vmHRV and vmHRV reactivity is moderated by the type of stressful event (those requiring physical activity vs. those that do not), whereas the relationship of resting vmHRV and vmHRV recovery is moderated by the reactivity response (vmHRV decrease vs. increase during reactivity). While there is laboratory evidence on these hypotheses, there is no evidence in daily life. Our study adds to this literature by for the first time systematically investigating whether the predictions of the vagal tank theory hold in daily life. We hypothesized that resting vmHRV relates to vmHRV reactivity (H1a) and that physical activity during reactivity moderates this relationship (H1b) such that lower physical activity accompanying a stressful event relates to higher vmHRV reactivity (H1c). We also hypothesized that if vmHRV decreased during a stressful event, higher resting vmHRV predicts faster recovery to the resting vmHRV level (H2a). Further, if vmHRV increased during the stressful event, resting vmHRV positively relates to vmHRV recovery time: the time vmHRV remains above resting levels (H2b). Based on the results of this study, we derive recommendations for assessing the physiological stress response in daily life continuously with vmHRV in a way that allows existing theoretical frameworks of stress reactivity and recovery to be tested.

## Materials and methods

We devised a 4-day, intensive longitudinal, observational study of HRV and stress in daily life. We followed recommendations that HRV studies should be conducted as within-subject designs given high between-person variations and interactions that influence HRV (Quintana and Heathers, 2014). Data were collected between September 2020 and February 2021 using a combination of device-based, event-based, and time-based self-report assessments. This combination of assessment strategies enabled us to systematically distinguish vmHRV during resting, reactivity, and recovery and thus investigate the intraindividual dynamic unfolding of stress and recovery in daily life. This study is part of a larger research project for which we had obtained ethical approval (Number 2020-02-00002) from the Ethics Committee of the Faculty of Human Sciences, University of Bern.

### Population and sample

The population of interest in this study were university students (18 years or older). This population was selected because students experience stress regularly (Leppink et al., 2016). We excluded individuals (a) whose knowledge of German was insufficient for our study; (b) who did not own a smartphone with a data plan; (c) who had been diagnosed with a sleep disorder, psychological disorder, cardiovascular disease, or metabolic disease; or (d) who were considered at high risk of contracting a severe case of COVID-19. Sleep disorders were an exclusion criterion related to a different research question of the research project. Psychological disorders and cardiovascular and metabolic diseases can influence HRV strongly and are thus recommended to be excluded unless they are characteristics of the target population of the study (Sammito et al., 2014). Being at high risk of contracting a severe case of COVID-19 was an exclusion criterion because this population was not allowed in the laboratory during data collection according to government regulations.

**Figure 2:**
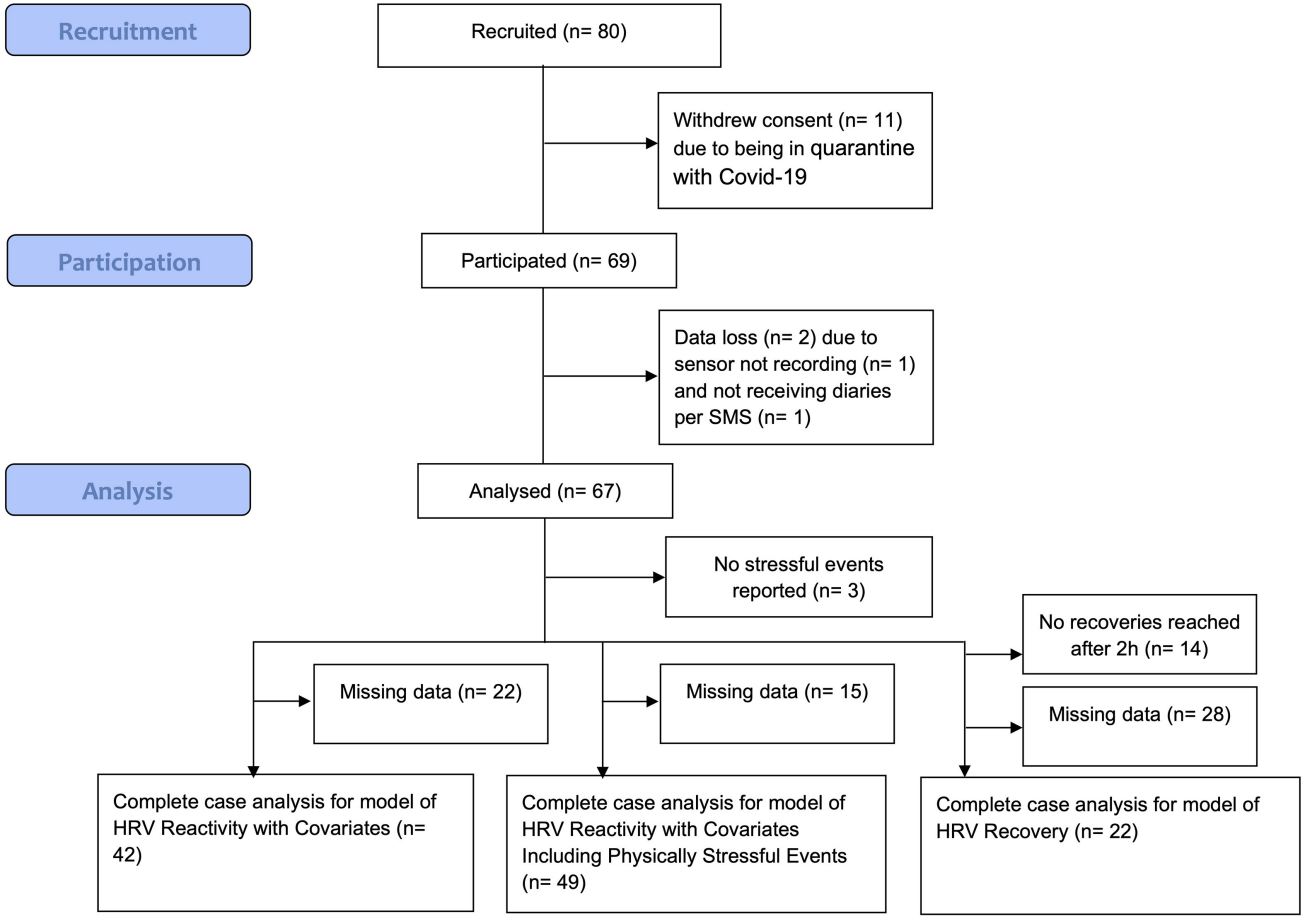
Flowchart to describe data loss. h, hours; HRV, heart rate variability. Data loss was also due to measurement failures of the electrocardiogram device. For the model of HRV recovery, data loss was further due to not being able to assign reported durations of stressful events reported in the bihourly diaries to the self-marked stressful events (mismatch). Recommendations for future studies to overcome these sources of data loss are made in the discussion.

The sample size was calculated a priori for a separate research question of the project, which focused on individual differences. Participants (*N* = 80) were recruited, but 11 people withdrew their consent to participate, mostly due to being in quarantine with COVID-19. Further, two participants were excluded either because the sensor did not record data or because the self-report questionnaires were not received. See Figure 2 for a flowchart visualizing this. This left 67 participants. Sample characteristics can be viewed in Table 1.

**Table 1:** Sample characteristics.

	**Mean**	**SD**	**Median**	**Minimum**	**Maximum**
BMI	23.3	22.6	22.6	19.4	33.2
MVPA (hours/week)	5.4	3.1	4.2	0	14
Chronic stress	18.8	7.0	17	7	39
Age	23.8	4.2	23	19	46
	**n (%)**				
**Biological sex**					
Female	53 (79%)				
Male	14 (21%)				
**Educational degree**					
Matura	67 (100%)				
**Employment**					
Employed	8 (12%)				
In education	57 (85%)				
Unemployed	2 (3%)				
**Monthly income**					
Up to 2,000 CHF	7 (10%)				
Btw. 20,001 and 4,000 CHF	15 (22%)				
Btw. 40,001 and 6,000 CHF	8 (12%)				
Btw. 60,001 and 8,000 CHF	11 (16%)				
Btw. 80,001 and 10,000 CHF	14 (21%)				
>10,000 CHF	9 (13%)				

*BMI, body mass index; MVPA, hours of moderate to vigorous physical activity during the last month; chronic stress, measured with the German Perceived Stress Scale; CHF, Swiss francs*.

### Measures

In this study, vmHRV during resting, reactivity, and recovery was calculated with data from an electrocardiogram, which was recorded continuously during waking hours with a physiological ambulatory monitoring device, the EcgMove 4. This sensor is optimized for the use in scientific studies and ambulatory assessment; it is lightweight (26 g), small (62.3 × 38.6 × 11.5 mm), records at a sampling rate of 1,024 Hz and a resolution of 12 bits and was worn with a chest belt including dry electrodes (Movisens Ltd., 2020). The influence of the parasympathetic nervous system on vmHRV was calculated with an established time domain measure, RMSSD (root mean square of the successive differences) (Laborde et al., 2018). In the time-based bihourly diary, we asked participants to report details on the experienced stressful events (e.g., duration), situational covariates (e.g., smoking) and other variables for the other parts of the project. See Table 2 for items used in this study.

**Table 2:** Questionnaire.

**Construct**	**Item wording**	**Response options**
Stressful events, first diary of the day (8 am)	Have you experienced one or more stressful events in the last 2 h?	No (0); yes, one stressful event (1); yes, two (2); yes, three (3); yes, four (4); yes, five (5)
Stressful events	Have you experienced one or more stressful events since the last interview?	No (0); yes, one stressful event (1); yes, two (2); yes, three (3); yes, four (4); yes, five (5)
Time of stressful event	Approximately when did the stressful event begin? (Please specify time e.g., 10:35)	hh:mm
Duration of stressful event	Approximately how many minutes did this stressful event last? (Please indicate in minutes e.g., 25, if the event is still ongoing, please estimate total duration).	Open
Alcohol consumption	Did you drink alcohol in the last 2 h?	No (0); yes (1)
Caffeine consumption	Did you drink any caffeinated drinks (e.g., coffee or energy drinks) in the last 2 h?	No (0); yes (1)
Nicotine consumption	Did you smoke cigarettes in the last 2 h?	No (0); yes (1)
Ambient noise, first diary of the day (8 am)	Have you been exposed to very loud ambient noise in the last 2 h?	No (0); yes (1)
Ambient noise	Have you been exposed to very loud ambient noise since the last interview?	No (0); yes (1)
Anticipated stress	I anticipate the next 2 h to be stressful.	No (0); yes (1)

*The items about time and duration of stressful events were used to calculate the start of recovery based on participants' retrospective estimate when it happened, to identify the right marker, and how long the self-marked stressful event lasted. The original German items can be seen in Supplementary Table 1*.

#### Stressful events

Participants were instructed to tap on the sensor when they started to feel stressed, marking this point in time as a stressful event (see Figure 1). The markers set by participants, were inspected for validity. Stress markers were excluded from analysis if they were (a) set when the sensor was not worn, (b) set in the same 5-min segment as an earlier marker, (c) specified by participants in the bihourly surveys as set by accident, or (d) related to stressful events lasting <5 min. At the data analysis stage, we noticed that participants had mainly marked psychologically stressful events accompanied by low physical activity (i.e., psychologically stressful events). To increase the range of physical activity, we included device-measured stressful events characterized by moderate to high level of physical activity (i.e., physically stressful events), for which we located segments with ≥ 3 METs (metabolic equivalent of tasks) for a duration of at least 5 min, to test hypotheses H1a-c thoroughly. The cut-off of ≥ 3 METs was used following the definition of the American College of Sports Medicine (ACSM) and the American Heart Association (Haskell et al., 2007).

#### Resting vmHRV

From the vmHRV data, 5-min segments were calculated complying with traditional recommendations of minimum segment length (Task Force, 1996). Resting vmHRV was measured with RMSSD and calculated as the mean of the 5-min segment preceding the minute in which the stress marker was set or physical activity was detected. Resting vmHRV was log-transformed to normalize distribution. Resting vmHRV was group mean centered to reflect the intraindividual change in resting vmHRV compared to the person's typical resting vmHRV. Group-mean centering involves subtracting the individual's group mean (in this case mean of all observed resting HRV values from one individual) from the individual's score (Bolger and Laurenceau, 2013).

#### VmHRV reactivity

To test the different hypotheses, vmHRV reactivity was operationalized in two ways, once representing the dependent variable (continuous vmHRV reactivity, hypotheses H1a-c), and once representing the dichotomous moderator variable (vmHRV reactivity increase vs. decrease, hypotheses H2a-b). As a basis, vmHRV measured with RMSSD during a stressful event (reactivity vmHRV) was calculated as the mean of the 5-min segment following the minute in which the stress marker was set or physical activity was detected. Although reactivity could potentially be longer than 5 min, it is essential for HRV segments to have the same length when comparing them (Task Force, 1996). To estimate vmHRV reactivity as the dependent variable (H1a-c), the difference between reactivity and resting vmHRV was calculated by subtracting resting from reactivity vmHRV; this difference indicated how much vmHRV changed after marking a stressful event compared to resting. To investigate the moderating effect of vmHRV reactivity and resting vmHRV on recovery (H2a-b), vmHRV reactivity was dummy-coded, with 0 signifying a decrease and 1 an increase during reactivity compared to resting levels. Note that there was no case with identical resting and reactivity vmHRV.

#### VmHRV recovery

VmHRV recovery, measured using the parameter RMSSD, was calculated as the time in minutes after the stressful event until vmHRV returned to resting levels (see Figure 1). We did this in two steps: locating the end of the stressful event (Step 1), and calculating the time in minutes after the stressful event (Step 2). In Step 1, we located the end of the stressful event (i.e., the start of the recovery phase) based on participants' retrospective estimate in the diary. For each event that had occurred since the last diary, participants indicated, how long the event lasted, and at what exact time it occurred (see items in Table 2). Then, we added the self-reported duration to the self-marked beginning of the stressful event. This was done using the following rules: (a) if multiple markers were found around the time of the stressful event indicated in the diary, the closest marker to that time was chosen. (b) If no marker was found 2 h around the time indicated in the diary, no recovery phase was calculated. In Step 2, we calculated the time in minutes after stressful events until vmHRV returned to resting vmHRV levels prior to the stressful event. To comply with the requirement to use same-length segments when investigating stress response processes, we did this in 5-min segment steps. For example, if vmHRV resting levels were not reached in the first 5-min segment after the end of the stressful event, we checked the next 5-min segment shifted by 1 min and so forth, until resting vmHRV was reached. If vmHRV did not return to the vmHRV resting level before a subsequent stressful event was marked or more than 120 min passed, recovery was recorded as not reached.

#### Physical activity

The amount of physical activity that accompanied a stressful event was operationalized as MET, computed from triaxial accelerometer data measured by the EcgMove 4 sensor. The physical activity requirement of a stressful event (H1a-c) was operationalized as the mean MET during the 5-min resting vmHRV segment (resting MET), the 5-min vmHRV reactivity segment (reactivity MET) and during the recovery phase (i.e., after the stressful event ended until vmHRV recovery was reached) (recovery MET). Physical activity is an important covariate of HRV in ambulatory settings; consequently, it is vital to adjust for physical activity because it can affect HRV greatly (Malik and Camm, 1995). Improbable values, that we defined as three times higher than the mean of functional capacity in exercise testing (> 79.8 MET per minute; Jetté et al., 1990), were excluded from analyses.

#### Covariates

We measured several covariates following recommendations and guidelines for studies assessing HRV and physiological stress (Pieper et al., 2010; Kudielka et al., 2012; Wells et al., 2012; Sammito et al., 2014; Laborde et al., 2017). We additionally created directed acyclical graphs (Pearl, 1995; Rohrer, 2018) to identify further plausible covariates based on the available literature (see Supplementary Figures 1, 2). These included breathing pace and supine body position measured by the EcgMove 4 sensor; alcohol, caffeine, and nicotine consumption; exposure to ambient noise; and anticipation of stress measured by self-report in the bihourly short surveys (see Table 2). Further, chronic stress was measured with the validated German Perceived Stress Scale (PSS-10, Schneider et al., 2020) because this can influence HRV (Kim et al., 2008) or might make awareness of separate stressful events more difficult. The reliability of the PSS-10 was excellent (α = 0.91). Additionally, body mass index (BMI) calculated from objective height and weight measured during the baseline laboratory appointment, and moderate to vigorous physical activity during the preceding month as an indicator of physical fitness were assessed with the International Physical Activity Questionnaire (IPAQ). These were measured because both can influence HRV (Sammito et al., 2014). Supine position during resting, reactivity, and recovery phase was calculated as minutes of the time segment spent in supine position. When anticipated stress or alcohol, caffeine, or nicotine consumption were reported, the previous two and subsequent 2 h were dummy coded as the factor being present. For ambient noise, only the preceding 2 h were dummy coded.

### Procedure

Participants were recruited *via* mailing list of the University of Bern, a platform of the Institute of Psychology that credits hours as a trial subject, and the website of the Department of Health Psychology and Behavioral Medicine. When registering for the study, participants were asked to send their smartphone numbers to the research team to receive daily diary prompts during the study. After registering for the study, participants received an email with a confirmation of their two laboratory appointments, study information and consent form, instructions concerning COVID-19 safety measures, and instructions to prepare for the first laboratory appointment. Participants were instructed to (a) shave excess body hair, and not use body lotion in the chest area where the tracker was to be worn during the study; (b) not to engage in intense physical activity 24 h before their first appointment; and (c) not to engage in any physical activity, eat, or consume caffeine 2 h before the first laboratory appointment (Laborde et al., 2017; Movisens Ltd., 2020). In the week before the study, participants received a reminder of their appointment and of the instructions. During the first laboratory appointment on Monday morning, participants handed in their signed consent forms. A research assistant then helped them put on the chest belt with an EcgMove 4 sensor, demonstrating how and where to wear it correctly. Then, participants completed a 20-min questionnaire containing questions about sociodemographic variables and covariates and participated in a traditional baseline HRV measurement. Participants were instructed with implementation intentions (Gollwitzer, 1999, in our case: “if I feel stressed, then I tap on the sensor”) to report stressful events by tapping on the sensor when they start to feel stressed. Implementation intentions have proved successful in modifying behavior (Hagger and Luszczynska, 2014). Participants then wore the EcgMove 4 sensor during waking hours except during showers and swims for 4 days until their second laboratory appointment. During this time, participants also answered bihourly prompted surveys, 8 per day, about the duration and marked stressful events and covariates. As part of the larger research project, participants also got prompted to engage in short stress reduction exercises. During the second laboratory appointment on Friday afternoon, participants again completed a 20-min questionnaire, engaged in another traditional baseline HRV measurement, and returned their EcgMove 4 sensors. Participants who wished to do so received a summary of their accelerometer and electrocardiogram. Psychology students were also offered course credit. The amount of course credit was calculated according to the amount of time that participation took. This is a usual measure of compensation for the University of Bern.

### Data analysis

#### Data preparation

Based on the raw electrocardiogram signal, the Movisens Data Analyzer software version 1.13.7 (Movisens Ltd., 2019) performed artifact removal. If amplitude and number of zero crossings per second were not within normal physiological range, the measurement was removed. In addition, the software uses an adapted algorithm from Clifford et al. (2002) to check for valid changes of consecutive RR-intervals and R-peak amplitudes. After artifact removal, we used the DataAnalyzer Software by Movisens to aggregate the data to 60 second resolution for data analysis. The rest of the data preparation was conducted with IBM SPSS Statistics (Version 25, IBM Corp, 2017). Missing self-report data in intensive longitudinal studies are often interpreted as missing at random (Bolger and Laurenceau, 2013), a strategy that has also been supported by research (e.g., Sun et al., 2020). Physiological data missing from the EcgMove 4 sensor are likely missing at random due to movement artifacts. Moreover, missingness exceeded 40%, meaning that data imputation was not recommended (Jakobsen et al., 2017). Consequently, missing data were deleted from analysis list-wise.

For all measures, outliers diverging more than three standard deviations from the mean were replaced with the value three standard deviations from the mean to approach them to the distribution (Tabachnick et al., 2019). RMSSD was log-transformed to adjust for skewed distribution (lnRMSSD). Self-reported physical activity was converted into hours, time spent in the study was converted into days, and self-reported chronic stress was standardized. All covariates except chronic stress were grand mean centered to facilitate interpretation of the results.

#### Hypotheses testing

The data were analyzed in RStudio (R Core Team, 2021) with the lme4 package (Bates et al., 2015) using multilevel modeling for repeated-measures data to accommodate the nested data structure. Within-subject causal processes were modeled for the hypotheses (Bolger and Laurenceau, 2013). Time passed since having started the study was included in the models, which is recommended for unevenly spaced data points when working with real-time momentary data (Schwartz and Stone, 2007). Inclusion of breathing pace as a covariate was not possible in any of the models because too many data were missing due to movement artifacts distorting the amplitudes of the electrocardiogram. Further, nicotine consumption could not be added as a covariate because this population reported nicotine consumption very rarely and models did not converge.

To test whether higher resting vmHRV relates to more adaptive reactivity to stressful events in daily life (H1a-c), reactivity vmHRV, the continuous intraindividual difference between reactivity and resting vmHRV, was predicted in a linear mixed model including resting vmHRV as the independent variable and the covariates (see methods section). Because participants mainly marked stressful events accompanied by low physical activity as indicated by MET (*M* = 1.74 MET, *SD* = 1.34), we ran an exploratory model additionally including physically stressful events.

To test whether higher resting vmHRV related to more adaptive recovery from stressful events in daily life (H2a-b), vmHRV recovery was predicted in a generalized linear mixed model with Poisson distribution. Estimates in this model are reported as incidence rate ratios with values greater than one being interpreted as the percentage increase and values smaller than one as the percentage decrease for a one unit increase in the predictor variable (Atkins et al., 2013). In this model, vmHRV recovery (in minutes) was the dependent variable, and independent variables included resting vmHRV, vmHRV reactivity (0 = decreased vs. 1 = increased vmHRV during reactivity), and the interaction between resting vmHRV and vmHRV reactivity. Covariates included were resting MET, MET during recovery phase, time passed since having started the study. No further covariates could be entered, as otherwise the model failed to converge.

## Results

Participants each set between 0 to 36 markers to report stressful events over the 4 days of the study (*M* = 8.0, *SD* = 5.9). Self-reported duration of stressful events ranged from 1 to 240 min (*M* = 21, *SD* = 30 min). A pile-up of stressful events occurred during three recoveries, meaning that additional stressful events were marked before recovery was reached, equaling 1.7% of recoveries. Further, 12 recoveries (6.6%) were not reached after 120 min and were thus excluded from analysis. Characteristics of stressful events can be seen in Table 3.

**Table 3:** Self-marked stressful event characteristics.

**Variables**	**Mean**	**SD**	**Median**	**Min**	**Max**
Resting RMSSD (ms)	40.74	23.79	35.52	4.05	183.99
Reactivity RMSSD (ms)	43.12	29.57	37.26	2.98	285.97
RMSSD Reactivity (ms)	2.42	1.38	19.27	−99.15	101.99
RMSSD recovery time (min)	15.67	22.47	5.50	0.00	111.00
**MET**					
Resting	1.6	0.7	1.4	1	4.8
Reactivity	1.7	1.3	1.0	1	6.5
Recovery	1.6	0.4	1.6	1.3	4.8
**Supine position (minutes)**					
Resting	0.1	0.6	0	0	4
Reactivity	0.2	0.8	0	0	5
Recovery	0.3	0.4	0.2	0	1.5
	**n (%)**				
Anticipated stress (experienced)	40 (27%)				
Ambient noise (experienced)	12 (8%)				
Caffeine (consumed)	39 (27%)				
Alcohol (consumed)	6 (4%)				
Nicotine (consumed)	11 (8%)				

*HRV, heart rate variability; RMSSD, root mean square of the successive differences; MET, metabolic equivalent of task; ms, milliseconds; min, minutes*.

For the hypotheses concerning vmHRV reactivity, the total analysis data set consisted of 197 observations (i.e., self-marked stressful events) from 42 participants. In the model including the physically stressful events derived from METs, 490 observations from 49 participants were included. For the hypotheses concerning vmHRV recovery, the analysis data set consisted of 57 observations from 22 participants for the model without covariates. An overview of the number of participants included in each model can also be seen in Figure 2.

### Relationship between resting heart rate variability and reactivity

**Figure 3:**
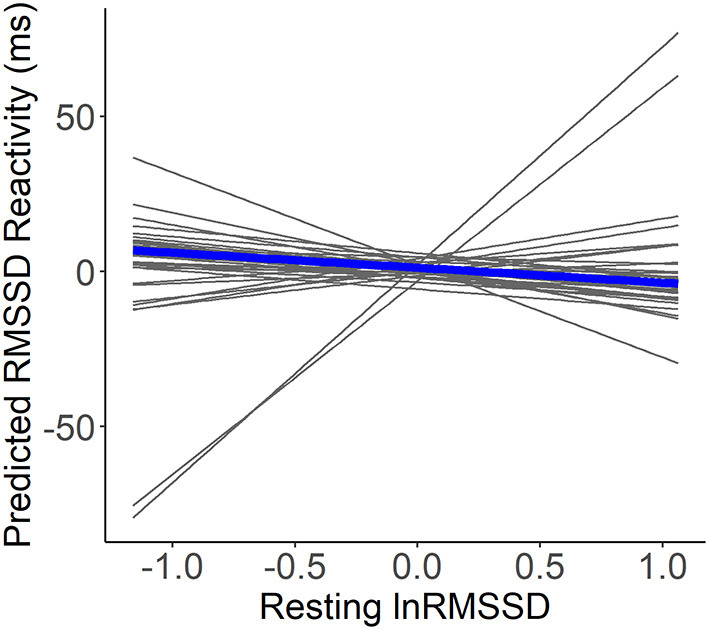
Within-person associations of resting lnRMSSD and predicted RMSSD reactivity. lnRMSSD, root mean square of the successive differences (log-transformed); ms, milliseconds. Association between group mean centered resting lnRMSSD and predicted RMSSD reactivity with the thick line representing mean within-person association compared to individual associations (thin lines). Absolute magnitude of RMSSD can be seen in Table 3.

For the model containing only self-marked stressful events, visual inspection suggested that higher resting lnRMSSD was not related to RMSSD reactivity (see Figure 3). Further, contrary to hypothesis H1a-c, resting lnRMSSD did not relate to RMSSD reactivity and reactivity MET did not moderate this relationship (see Table 4). In terms of covariates, there was a main effect of resting and reactivity MET as well as supine position. Every 1-unit increase in reactivity MET related to an RMSSD reactivity decrease of 5.8 milliseconds. Further, every 1-unit increase in resting MET and 1-min increase in reactivity supine position related to an RMSSD reactivity increase of 4.9 milliseconds and of 6.6 milliseconds, respectively.

**Table 4:** Linear mixed model of RMSSD reactivity with covariates.

				**CI**_**95**_	
**Fixed effects (intercept, slopes)**	**Estimate**	**SE**	**p**	**LL**	**UL**
Intercept	0.93	1.64	0.572	−2.28	4.13
Resting lnRMSSD^a^	−5.15	2.97	0.083	−10.98	0.68
Resting lnRMSSD^a^*Reactivity MET	−0.27	2.69	0.919	−5.00	5.55
Resting MET	4.90	2.17	0.024	0.66	915
Reactivity MET	−5.82	1.74	0.001	−9.22	−2.41
Resting supine^b^	−1.44	4.36	0.742	−9.97	7.10
Reactivity supine^b^	6.59	3.27	0.044	0.18	13.00
Time in study	1.25	0.88	0.156	−0.48	2.98
Caffeine	0.81	2.66	0.759	−4.39	6.02
Alcohol	−2.78	5.65	0.623	−13.85	8.30
Anticipated stress	−1.37	2.28	0.548	−5.83	3.09
Ambient noise	−1.93	4.36	0.658	−10.47	6.61
MVPA	0.19	0.40	0.639	−0.59	0.96
BMI	−0.14	0.42	0.745	−0.96	0.69
Chronic stress^c^	−0.27	1.38	0.842	−2.98	2.43
**Random effects ([co-]variances)**	**Variance**	**Std. dev**.			
**Level 2 (between person)**					
Intercept	14.38	3.79			
**Level 1 (within person)**					
Residual	191.25	13.83			

*N, 197 observations by 42 participants; lnRMSSD, root mean square of the successive differences (log-transformed); MET, metabolic equivalent of task; time in study, time in days that participants have been in the study at the point of a stressful event; MVPA, hours of moderate to vigorous physical activity; BMI, body mass index; chronic stress, measured with the German Perceived Stress Scale; CI_95_, 95% confidence interval; LL, lower limit; UL, upper limit of confidence interval*.

a *Resting lnRMSSD was group mean centered,*

b *Supine position corresponds to minutes spent in supine position during resting or reactivity,*

c *Chronic stress was standardized*.

**Figure 4:**
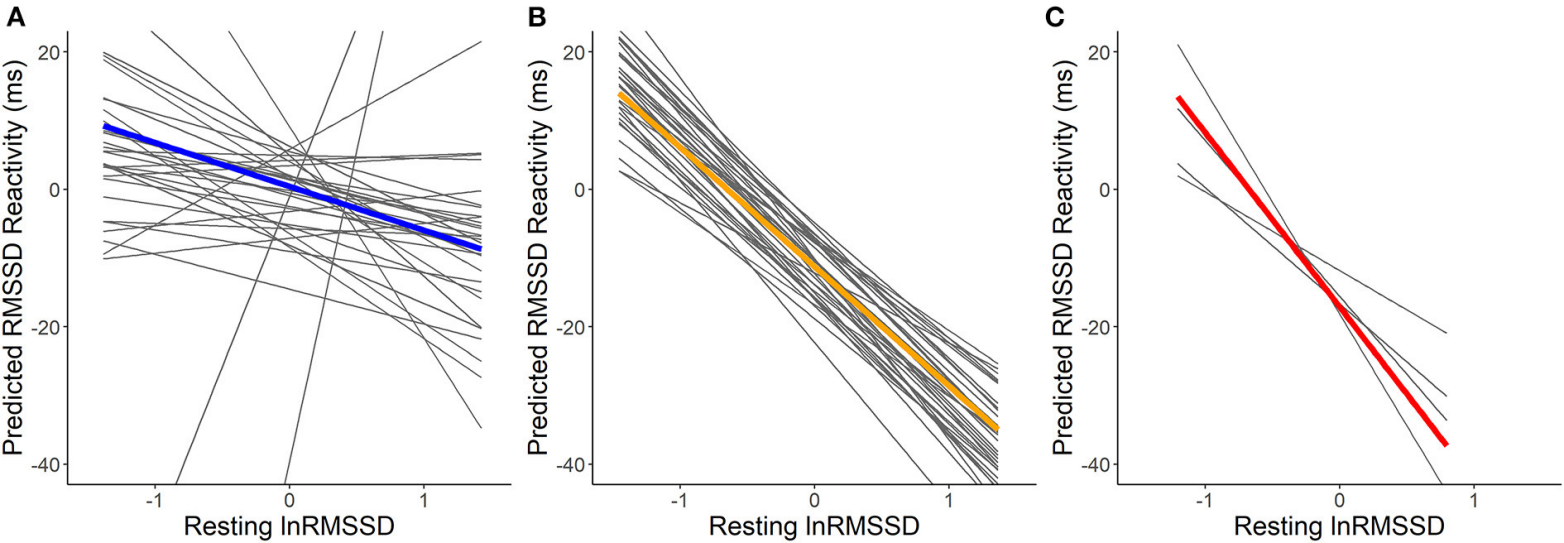
Within-person association of resting lnRMSSD and predicted RMSSD reactivity including physically stressful events. lnRMSSD, root mean square of the successive differences (log-transformed); ms, milliseconds. Association between group mean centered resting lnRMSSD and predicted RMSSD reactivity for events with physical activity of light [MET <3; **(A)**], moderate [MET > 3 <6; **(B)**] and vigorous intensity [MET > 6; **(C)**]. Thick lines represent mean within-person association compared to the individual associations (thin lines). Absolute magnitude of RMSSD can be seen in Table 3.

For the model additionally containing physically stressful events, visual inspection of Figure 4 suggests that higher resting lnRMSSD was related to lower RMSSD reactivity. Confirming hypothesis H1a, resting lnRMSSD related significantly to RMSSD reactivity (see Table 5). Every 1 unit increase in resting lnRMSSD related to an RMSSD reactivity decrease of 14 milliseconds. Confirming H1b and H1c, reactivity MET moderated this relationship in the hypothesized direction. Lower reactivity MET related to higher RMSSD reactivity. We also found a main effect of reactivity MET, where a 1-unit increase in reactivity MET related to an RMSSD reactivity decrease of 3.6 milliseconds. For covariates, every additional day of enrolment in the study related to an RMSSD reactivity increase of 1.4 milliseconds and having consumed alcohol related to an RMSSD reactivity decrease of 5 milliseconds.

**Table 5:** Linear mixed model of RMSSD reactivity with covariates including physically stressful events.

				**CI**_**95**_	
**Fixed effects (intercept, slopes)**	**Estimate**	**SE**	**p**	**LL**	**UL**
Intercept	−7.06	1.29	<0.001	−9.59	−4.52
Resting lnRMSSD^a^	−14.06	1.35	<0.001	−16.71	−11.41
Resting lnRMSSD^a^^*^Reactivity MET	−3.85	0.75	<0.001	−5–33	−2.38
Resting MET	0.34	0.87	0.696	−1.36	2.04
Reactivity MET	−3.55	0.37	<0.001	−4.27	−2.83
Resting supine^b^	2.13	3.50	0.542	−4.72	8.99
Reactivity supine^b^	6.69	3.75	0.075	−0.67	14.05
Time in study	1.43	0.44	0.001	0.57	2.29
Caffeine	0.14	1.56	0.931	−2.92	3.19
Alcohol	−4.98	2.37	0.036	−9.64	−0.33
Anticipated stress	0.03	1.33	0.981	−2.58	2.64
Ambient noise	−2.63	2.13	0.218	−6.82	1.55
MVPA	0.20	0.35	0.575	−0.49	0.89
BMI	0.22	0.42	0.594	−0.60	1.04
Chronic stress^c^	−0.26	1.21	0.826	−2.63	2.10
**Random effects ([co-]variances)**	**Variance**	**Std. dev**.			
**Level 2 (between person)**					
Intercept	36.87	6.07			
**Level 1 (within person)**					
Residual	147.93	12.16			

*N, 490 observations by 49 participants; lnRMSSD, root mean square of the successive differences (log-transformed); MET, metabolic equivalent of task; time in study, time in days that participants have been in the study at the point of a stressful event; MVPA, hours of moderate to vigorous physical activity; BMI, body mass index; chronic stress, measured with the German Perceived Stress Scale; CI_95_, 95% confidence interval; LL, lower limit; UL, upper limit of confidence interval*.

a *Resting RMSSD was log-transformed and group mean centered*.

b *Supine position corresponds to minutes spent in supine position during resting or reactivity*.

c *Chronic stress was standardized*.

### Relationship between resting heart rate variability and recovery

**Figure 5:**
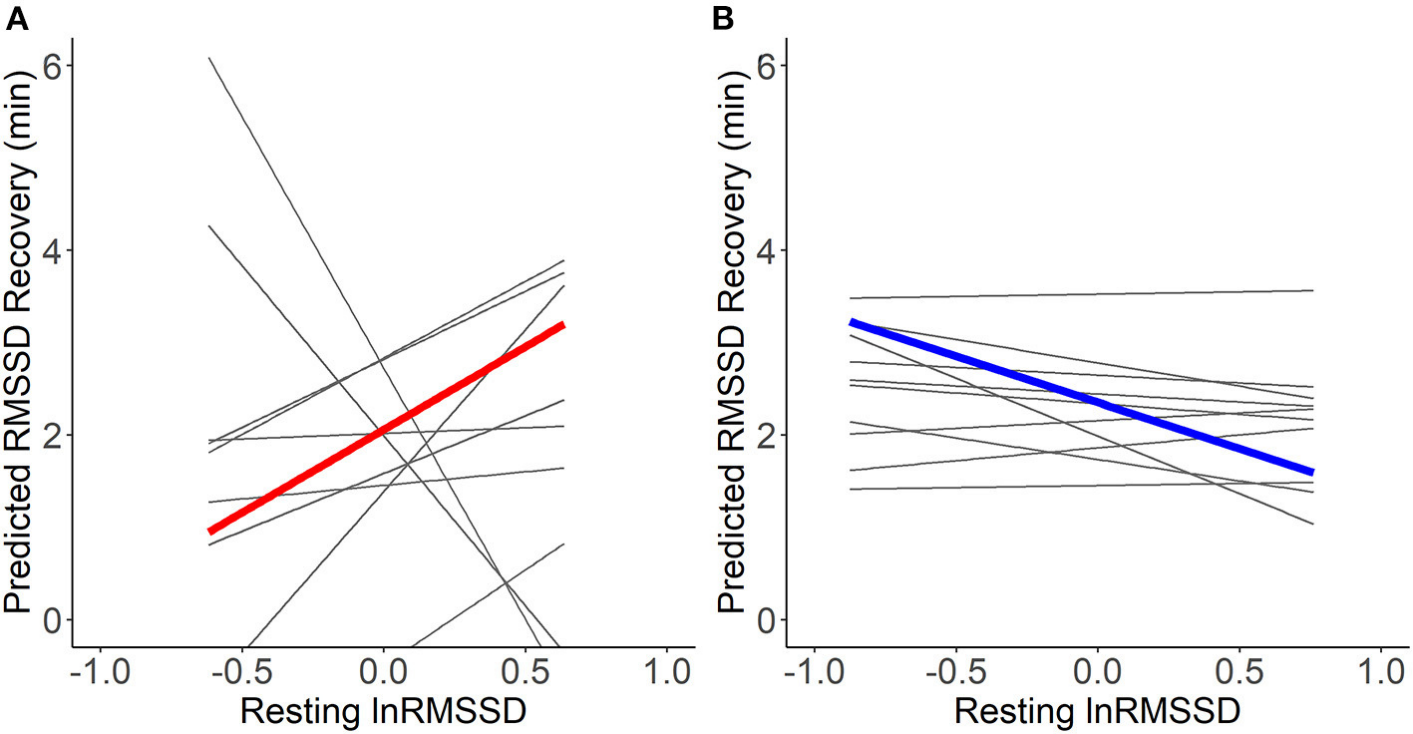
Within-person association of resting lnRMSSD and predicted RMSSD recovery. lnRMSSD, root mean square of the successive differences (log-transformed); ms, milliseconds; min, minutes. Association between group mean centered resting lnRMSSD and predicted RMSSD recovery for events where RMSSD decreased **(A)** vs. increased **(B)** during stressful events. Thick lines represent mean within-person association compared to the individual associations (thin lines). Absolute magnitude of RMSSD can be seen in Table 3.

Visual inspection of Figure 5 suggests that resting HRV was not related to more adaptive HRV recovery. Contrary to hypotheses H2a and H2b, higher resting lnRMSSD seemed to relate to slower recovery when RMSSD decreased during the stressful event and faster recovery when RMSSD increased during the stressful event. The statistical tests of the hypotheses presented in Table 6 confirm this. Every 1 unit increase in resting lnRMSSD related to a 4.3% increase in RMSSD recovery. RMSSD reactivity moderated the intraindividual association between resting lnRMSSD and RMSSD recovery, but not in the hypothesized direction. Finally, every 1-unit increase in resting MET related to a 1.4-min increase in RMSSD recovery.

**Table 6:** Generalized linear mixed model of RMSSD recovery.

				**CI**_**95**_	
**Fixed effects (intercept, slopes)**	**IRR**	**SE**	**p**	**LL**	**UL**
Intercept	8.72	2.82	<0.001	4.63	16.42
Resting lnRMSSD^a^	4.30	1.15	<0.001	2.55	7.26
Resting lnRMSSD^a^^*^RMSSD Reactivity	0.24	0.09	<0.001	0.12	0.50
RMSSD Reactivity^b^	0.88	0.10	0.235	0.71	1.09
Resting MET	1.44	0.14	<0.001	1.19	1.74
Recovery MET	1.11	2.53	0.962	0.01	94.75
Time in study	1.02	0.05	0.642	0.93	1.12
**Random effects ([co-]variances)**	**Variance**	**Std. dev**.			
**Level 2 (between person)**					
Intercept	2.03	1.43			

*N, 57 observations by 22 participants; lnRMSSD, root mean square of the successive differences (log-transformed); MET, metabolic equivalent of task; time in study, time in days that participants have been in the study at the point of a stressful event; IRR, incidence rate ratios; CI_95_, 95% confidence interval for incidence rate ratios; LL, lower limit; UL, upper limit of confidence interval*.

a *Resting lnRMSSD was log-transformed and group mean centered,*

b *RMSSD reactivity was dummy-coded*.

## Discussion

This study investigated whether vagal tank theory's predictions about adaptive stress response extend to daily life. Working from the assumptions of the vagal tank theory, we investigated the temporal dynamics of physiological stress measured by vmHRV during resting, reactivity, and recovery around stressful events in daily life. We hypothesized that higher resting vmHRV predicts more adaptive vmHRV reactivity to stressful events in daily life. This was only supported by the data if physically stressful events were considered additionally to perceived stressful events. In those analyses, we found that resting vmHRV relates to vmHRV reactivity and that reactivity MET moderates the relationship as hypothesized: VmHRV reactivity was lower when stressful events required physical activity. We further hypothesized that higher resting vmHRV predicts more adaptive vmHRV recovery from stressful events in daily life. This was not supported by the data. Contrary to hypotheses, we found that higher resting vmHRV relates to slower recovery when vmHRV decreased during the stressful event and faster recovery when vmHRV increased during the stressful event. A summary of the hypothesis and our results compared to other studies looking at high-resolution temporal vmHRV dynamics can be seen in Table 7.

**Table 7:** Overview of results.

**Hypothesis**	**Results**	**Compared to other studies**
Resting vmHRV relates to vmHRV reactivity (H1a)	Not confirmed in the model only including self-marked stressful events. Confirmed in model additionally including physically stressful events	In line with previous studies: Resting vmHRV related to reactivity vmHRV in the lab, in situations requiring physical activity (Review of studies looking at physical activity exercises: Stanley et al., 2013). Not in line with previous studies: Resting vmHRV related to reactivity vmHRV in the lab, in situations relying mainly on executive functions and not physical activity (Stroop tasks combined with concurrent auditory distractors: Spangler and McGinley, 2020; Selective attentional task with varying levels of cognitive load: Park et al., 2014; Working memory test: Hansen et al., 2003).
Physical activity during reactivity moderates this relationship (H1b)	Not confirmed in the model only including self-marked stressful events. Confirmed in model additionally including physically stressful events	We are not aware of any studies that investigated this.
Such that lower physical activity accompanying a stressful event relates to higher vmHRV reactivity (H1c)	Not confirmed in the model only including self-marked stressful events. Confirmed in model additionally including physically stressful events	In line with previous studies: Higher resting vmHRV related to higher levels of reactivity vmHRV in the lab, in situations relying mainly on executive functions and not physical activity (Stroop tasks combined with concurrent auditory distractors: Spangler and McGinley, 2020; Selective attentional task with varying levels of cognitive load: Park et al., 2014; Working memory test: Hansen et al., 2003).
If vmHRV decreased during a stressful event, higher resting vmHRV predicts faster recovery to resting vmHRV level (H2a)	Not confirmed: if vmHRV decreased during a stressful event, higher resting vmHRV predicted slower recovery to resting vmHRV level	Not in line with previous studies: Higher resting vmHRV related to faster vmHRV in the lab, after situations where vmHRV decreased (Review of studies looking at physical activity exercises: Stanley et al., 2013). Higher resting vmHRV related to higher recovery vmHRV levels in the lab, after situations where vmHRV decreased (Working memory test: Hansen et al., 2003). Resting vmHRV did not relate to vmHRV during recovery in the lab, after situations where vmHRV decreased (Physical activity exercise: Javorka et al., 2002)
If vmHRV increased during a stressful event, higher resting vmHRV predicts slower recovery to resting vmHRV level (H2b)	Not confirmed: if vmHRV increased during a stressful event, higher resting vmHRV predicted faster recovery to resting vmHRV level.	We are not aware of any studies that investigated this.

*vmHRV, vagally-mediated heart rate variability*.

### Relationship between resting heart rate variability and reactivity

The first hypothesis, that higher resting vmHRV would relate to more adaptive vmHRV reactivity to stressful events in daily life, was only confirmed when models also incorporated physically stressful events (i.e., events accompanied by physical activity). One explanation for this finding may be the requirements of the stressful events in daily life. In laboratory research, stimuli are usually selected to either require executive functioning or physical activity (e.g., running on a treadmill). For example, stimuli used in laboratory settings that require executive function include tasks such as video game challenges (e.g., Li et al., 2019), or arithmetic mental stress (e.g., Pan and Li, 2007; Vuksanović and Gal, 2007), where participants sit in front of a laptop without moving. In contrast, stressful events experienced in daily life may mostly involve a mix of both executive functioning and physical activity. For example, a person might be stressed due to an upcoming exam, while running to catch the bus. Thus, while the daily life setting in our study likely led to observing stressful events that are meaningful to participants, they could differ in their requirements from typical stressors used in laboratory settings (e.g., executive functions or running on a treadmill). We found a main effect of resting vmHRV being associated to a decrease in vmHRV reactivity in the model that also incorporated physically stressful events. This was likely due to more physically stressful events (490) being included compared to self-marked stressful events (197). In physically stressful events we would expect this association of resting vmHRV being associated to a decrease in vmHRV reactivity according to vagal tank theory (Laborde et al., 2018).

### Relationship between resting heart rate variability and recovery

Concerning the recovery, the prediction that higher resting vmHRV relates to more adaptive vmHRV recovery from stressful events in daily life was not supported by the data of this study. Possibly, a pile-up of stressful events, meaning participants encounter an additional stressful event before recovering from the first one, makes it more difficult to analyze recovery after real-life stressful events. In laboratory settings, this can be controlled and avoided much more easily. Vagal tank theory should be extended to incorporate predictions about stress pile-up, for instance that successive stressful events without the chance to fully recover are not adaptive because of their negative impacts on mental and physical performance (Dhabhar, 2018). In addition, it is not clear if vmHRV recovery dynamics are in fact adaptive in daily life at all, where participants are free to engage in diverse coping (e.g., going for a walk or asking for support), and where context potentially plays a larger role than in laboratory settings. Further, we found a main effect of higher resting lnRMSSD being associated to an increase in vmHRV recovery time. More research is needed in this area to understand this main effect of higher resting lnRMSSD and to enable conclusions about the extent to which the vagal tank theory's predictions (Laborde et al., 2018) about vmHRV recovery translate into daily life.

### Covariates

Supine position during reactivity related to an increase in vmHRV reactivity. This effect has also been demonstrated in various laboratory studies (Mourot et al., 2004; Grant et al., 2009), and it is important because daily life studies often do not control for body position when measuring HRV (an exception being for example the study by Vrijkotte et al., 2000). Supine position as a covariate was not significant in the model additionally containing physically stressful events, which might be explained by supine position and physical activity often excluding each other.

We also found a main effect of physical activity during reactivity relating to a vmHRV reactivity decrease. Additionally, physical activity during resting related to an increase in vmHRV reactivity. This could be explained by regression to the mean, in this case if physical activity during resting was high it is likely to not again show high physical activity during the reactivity segment. This idea is underlined by the covariate not being significant in the model additionally containing physically stressful events, because this model included cases where physical activity was high during reactivity. Overall, the effect of physical activity during resting and reactivity on reactivity HRV again underlines the importance of measuring physical activity along HRV (Quintana and Heathers, 2014; Laborde et al., 2017).

In the model additionally containing physically stressful events, having consumed alcohol related to a decrease in vmHRV reactivity. This effect of alcohol consumption relating to a reduction in reactivity vmHRV has also been shown in laboratory settings (Vaschillo et al., 2008).

Finally, in the model additionally containing physically stressful events, every additional day of enrolment in the study related to a vmHRV reactivity increase. This may indicate a day-of-the-week effect on stress reactivity, which could be caused by the requirements of different stressful events varying across the week.

### Strengths

This study for the first time systematically tested the predictions of the vagal tank theory (Laborde et al., 2018) in daily life. Strengths of this ambulatory assessment approach include minimizing retrospective distortion, which is important when investigating fast-changing intraindividual processes (Mehl and Conner, 2011; Bolger and Laurenceau, 2013), and high ecological and external validity. Daily life might involve pile-up of stressful events, less restricted use of coping mechanisms such as physical activity and mobilizing social support, and increased variation of contexts, including social contexts; all of these elements make studying naturalistic stressful events in daily life more complicated but also introduce real-world contexts.

Another strength of this study is adding to the limited literature (e.g., Spangler et al., 2018) looking at the temporal dynamics of vmHRV in high temporal resolution in daily life. Using objective measures such as HRV to learn more about autonomic nervous system activity around stressful events can help establish the biological plausibility of the link between stress and negative health outcomes (Kubzansky et al., 2014). Measuring HRV also provides real-time data with high temporal resolution to capture and study daily stress dynamics (Rohleder, 2019; Schlotz, 2019). The multimodal assessment of vmHRV measurements coupled with bihourly self-reports allowed information to be sampled about psychological, physiological, behavioral, and contextual factors of the naturalistic stressful events. Pairing this assessment strategy with assessment of more stable interindividual characteristics and careful considerations during the creation of graphical causal models for observational data allowed a broad spectrum of covariates to be included and emphasized the importance of variables across time scales when investigating stress in daily life.

### Limitations

When measuring HRV, a multitude of other environmental, social, psychological, and physiological factors also need to be measured and controlled for. Deciding which factors to control for is complicated and should be well-justified, because controlling for colliding variables and mediators could shift the value of the causal impact of interest away from the estimation of a relationship and lead to bias (Rohrer, 2018). However, because so few data are available about the influences of factors on high-resolution HRV measurements and naturalistic stressors in daily life, the process of finding the appropriate factors to control for was fraught with uncertainty. To overcome this limitation in this study, we worked with graphical causal models for observational data (directed acyclical graphs, Rohrer, 2018), which can be seen in Supplementary Figures 1, 2. Still, important factors might have been missed in the analyses of stressful events.

The study's design also relied on self-marking and self-report to locate naturalistic stressful events and retrospectively estimate their duration. This required participants to be aware of stressful events and to recall their duration accurately. Small deviations in reporting could thus have affected the located segments for calculating vmHRV. Further, matching self-reported durations to stressful events with the help of participants' estimated time points was difficult due to discrepancies, which could be due to misremembering or not wanting to answer additional questions. Furthermore, the study's short duration and missing HRV data, possibly caused by movement artifacts or by participants' wearing the sensor incorrectly or not at all, led to a limited number of observed vmHRV recoveries. Participants may also have had varying definitions and thresholds for what they considered stressful, and the requirement to set a marker may even have provoked reactivity by bringing awareness to feelings of stress. However, self-marking of stressful events has advantages, such as events being likely personally relevant.

Also, this study used a very specific sample: Swiss students at the University of Bern, of a specific age group and with many female participants (*N* = 53; 79%). Although stress is prevalent in students (Leppink et al., 2016), this demographic might face distinct kinds of stressful events. The age group and biological sex are important to bear in mind, because both can also influence HRV (Sammito et al., 2014). Thus, this study's preliminary results cannot be generalized to other demographics.

Finally, as part of the wider study project, participants were asked to participate in brief stress reduction activities at randomized timepoints. This could have led to participants experiencing and reporting fewer stressful events or also to faster recoveries, if they engaged in the exercises during the recovery phase after a stressful event. Since the exercises were conducted at random time points, this should not have distorted the analysis. Engaging in relaxation exercises could also have helped to observe a wider variability in resting vmHRV, which could have helped the observation of the temporal dynamics of vmHRV in daily life.

### Recommendations for future studies of the physiological stress response with heart rate variability in daily life

Due to differences in laboratory compared to real life settings, there are necessary adaptations to be made when assessing the physiological stress response in daily life with vmHRV, thus enabling testing theoretical frameworks based on laboratory research. Such studies must be planned so that they include stressful events with varying requirements and both physiological and psychological covariates, some of which may be especially relevant in daily life contexts, such as physical activity, ambient noise, and anticipated stress. Our study allows for recommendations and guidelines for future studies of the temporal dynamics of the physiological stress response by measuring vmHRV in daily life. We summarized them in Table 8.

**Table 8:** Lessons learnt and recommendations for future studies.

**Area**	**Dimension**	**Recommendations**
Population and sample	Sample size	Use the observed frequency of stressful events in this study (during 4 days: *M* = 8.0, *SD* = 5.9) to inform a priori power analyses for future studies of the stress response in daily life.
	Population	Investigate other demographics that frequently experience stressful events: different demographics might face distinct kinds of stressful events in their lives and factors such as age and biological sex can have an influence on HRV dynamics
Study design	Duration	Experiment with longer study durations in order to better observe more stressful events and sufficient within-person variance for multilevel modeling
	Types of stressors	Include different kinds of stressful events (psychological, cognitive, physical activity)
HRV	Measurement	Use other measurement techniques such as sticky electrodes instead of chest belts to mitigate data loss
	Segmentation	Consider segmentation procedure that is more resistant to delays in reporting of stressful events (e.g., measurement of resting HRV in the morning instead of right before a stressful event)
	Segment length	Test different segment lengths in daily life because traditional 5-min segments might not be optimal for measuring HRV temporal dynamics in daily life
	Resting HRV	Use alternative resting HRV segments such as measuring resting HRV in the morning instead of right before a stressful event or using a traditional baseline resting HRV measurement recorded in the laboratory
Covariates	Respiratory rate	Use piloting to investigate if used monitors can accurately measure respiratory rate in ambulatory settings
	Supine positon	Control for supine position during HRV measurements
	Physical activity	Control for physical activity such as MET during HRV measurements
	Anticipated stress	Control for anticipated stress during HRV measurements
	Ambient noise	Objectively measure and control for ambient noise during HRV measurements

*HRV, heart rate variability; MET, metabolic equivalent of task*.

A valuable research question for a future study could also be to focus on the heterogeneity of the effects with a bigger sample, making the detection of random slopes possible. The results of this study can help conduct a power analysis to find the needed number of participants and observations for such a study.

Beyond observation, the effect of higher resting vmHRV on more adaptive vmHRV reactivity to stressful events in daily life might also be tested by experimentally manipulating resting vmHRV through, for example, mindfulness meditation (Libby et al., 2012) and inducing stress in daily life. This would allow some control over the demands of the stressful events instigated. Alternatively, real-time analysis of HRV data might allow stressful events in daily life to be detected through individually meaningful decreases of vmHRV controlled for physical activity with algorithms such as Schwerdtfeger and Rominger (2021) have used. Adapting such algorithms could maybe in the future allow to calculate vmHRV recovery time by incorporating the influence of physical activity instead of retrospectively controlling for it.

Additionally, studies should consider incorporating HRV parameters that vary time horizons when investigating HRV stress dynamics. For example, measuring resting vmHRV in the morning could be informative of overall recovery from the preceding day, possible accumulation of sleep-related fatigue across days, and readiness to manage stressful events during the day to come. Further, to improve the investigation of the relationship between resting vmHRV and vmHRV recovery, it might be helpful to have more frequent diaries (e.g., hourly surveys). This should improve matching the self-reported durations for the stressful events in the diaries to the self-marked stressful events, and thus prevent data loss.

Further studies also need to examine what factors influence HRV around stress in daily life and thus might need to be controlled for. Relying exclusively on results from laboratory studies for this is insufficient, because daily-life contexts vary much more than laboratory settings. Testing the generalizability of factors' influence on HRV in daily life makes for interesting future research questions and underlines the importance of environment and context. With more studies focusing on different types of stressors, time horizons, and contexts of naturalistic stressful events, theories might be extended to better understand what “adaptive” dealing with stress in everyday life actually looks like.

## Conclusion

The findings of this study shine some light on how people react to and recover from stressful events in daily life and possibly contribute to better understanding adaptive stress management. The significance of individual differences in resting vmHRV as possible predictors of adaptive stress response in daily life are highlighted, with implications for stress management strategies boosting HRV such as mindfulness meditation. We hope this study inspires further research into the dynamic unfolding of stress and recovery in everyday life according to different types of naturalistic stressors, varying time horizons, and context factors specific to everyday life.

## Data availability statement

The datasets presented in this study can be found in online repositories. The names of the repository/repositories and accession number(s) can be found at: Harvard Dataverse Repository [https://doi.org/10.7910/DVN/TUHFCD].

## Ethics statement

The studies involving human participants were reviewed and approved by Ethics Committee of the Faculty of Human Sciences, University of Bern. The patients/participants provided their written informed consent to participate in this study.

## Author contributions

MB and JI co-created the ideas described in this paper, developed and supervised the empirical experiments, and wrote the original draft of the text. MB wrote the analytic code and evaluated the data and JI double-checked their correctness. All authors critically revised and approved the final submitted version of the work.

## Funding

Open access funding was provided by the University of Bern.

## Conflict of interest

The authors declare that the research was conducted in the absence of any commercial or financial relationships that could be construed as a potential conflict of interest.

## Publisher's note

All claims expressed in this article are solely those of the authors and do not necessarily represent those of their affiliated organizations, or those of the publisher, the editors and the reviewers. Any product that may be evaluated in this article, or claim that may be made by its manufacturer, is not guaranteed or endorsed by the publisher.
