# Erratum: Physiological stress reactivity and recovery: Some laboratory results transfer to daily life

**DOI:** 10.3389/fpsyg.2023.1178216

**Published:** 2023-03-14

**Authors:** Frontiers Production Office

**Affiliations:** Frontiers Media SA, Lausanne, Switzerland

**Keywords:** stress, daily life, heart rate variability, vagal tank theory, stressful events, temporal dynamics, physiological stress response

An omission to the funding section of the original article was made in error. The following sentence has been added: “Open access funding was provided by the University of Bern.”

The original article has been updated.

