# Supplementary material for: Physiological stress reactivity and recovery: Some laboratory results transfer to daily life
Source: Front Psychol. 2022 Aug 15;13:943065. doi: 10.3389/fpsyg.2022.943065 (PMC9421134; doi:10.3389/fpsyg.2022.943065)
Supplement: Supplementary file 1 [file Data_Sheet_1.docx]

Physiological Stress Reactivity and Recovery: Some Laboratory Results Transfer to Daily Life

Melanie Bamert^1*^ & Jennifer Inauen^1^

^1^ Department of Health Psychology and Behavioral Medicine, Institute of Psychology, University of Bern, Bern, Switzerland

*** Correspondence:**Melanie Bamert
melanie.bamert@unibe.ch

Electronic Supplementary Material

Contents:

1. Directed acyclical graphs
2. Questionnaire in German

**1 Directed acyclical graphs**

**Figure S1.** Directed acyclical graph for hypothesis 1.

*
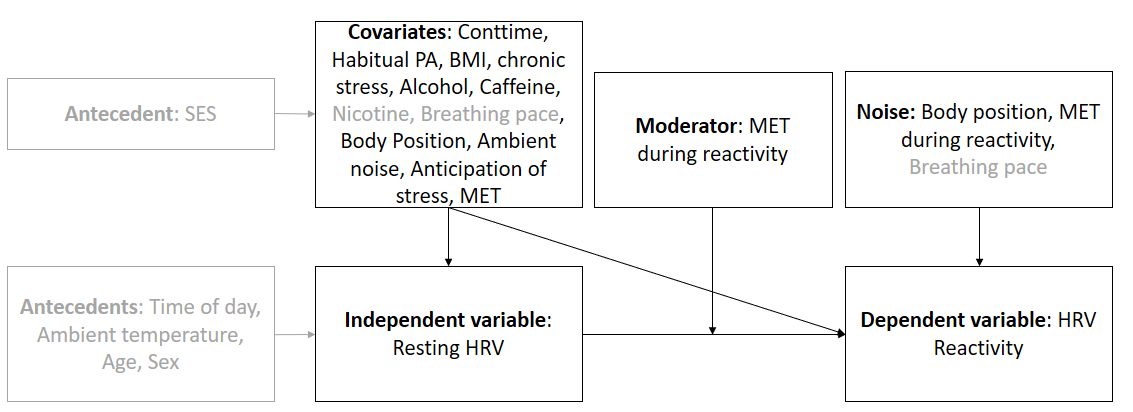
Note.* SES = socioeconomic status, conttime = time participants have been in study at time of a stressful event (continuous time), Habitual PA = moderate to vigoros physical activity, BMI = body mass index, chronic stress = measured with the German Perceived Stress Scale, MET = metabolic equivalent of task, HRV = heart rate variability. Grey means not included due to being antecedents or models not converging.


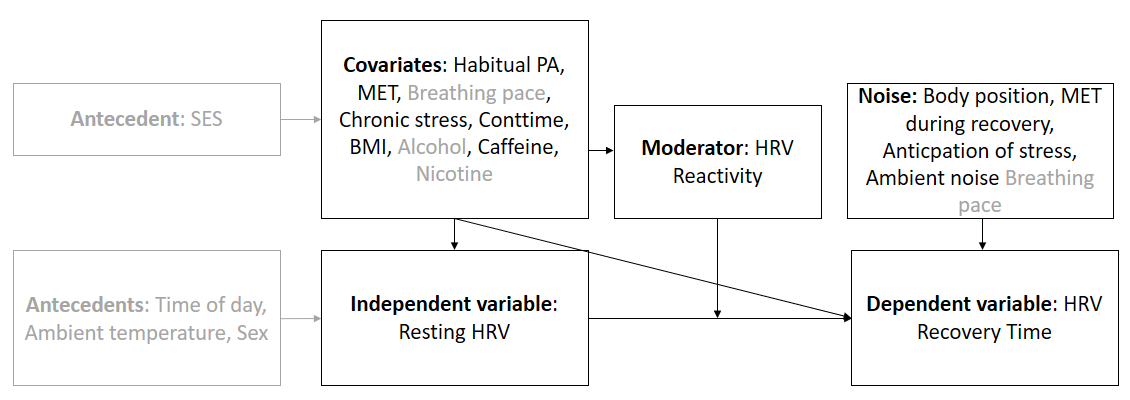
**Figure S2.** Directed acyclical graph for hypothesis 2.

*Note.* SES = socioeconomic status, conttime = time participants have been in study at time of a stressful event (continuous time), Habitual PA = moderate to vigoros physical activity, BMI = body mass index, chronic stress = measured with the German Perceived Stress Scale, MET = metabolic equivalent of task, HRV = heart rate variability. Grey means not included due to being antecedents or models not converging.

**2 Questionnaire in German**

Supplementary Table S1

*Questionnaire in German*

| *Construct* | *Item wording* | *Response options* |
| --- | --- | --- |
| Stressful events, first diary of the day (8 am) | Haben Sie in den letzten zwei Stunden ein oder mehrere stressige Ereignisse erlebt? | nein (0); ja, ein stressiges Event (1); ja, zwei (2); ja, drei (3); ja, vier (4); ja, fünf (5) |
| Stressful events | Haben Sie seit der letzten Befragung ein oder mehrere stressige Ereignisse erlebt? | nein (0); ja, ein stressiges Event (1); ja, zwei (2); ja, drei (3); ja, vier (4); ja, fünf (5) |
| Time of stressful event | Wann hat das stressige Ereignis etwa begonnen? (Bitte Uhrzeit angeben z.B. 10:35) | hh:mm |
| Duration of stressful event | Wie viele Minuten hat dieses stressige Ereignis etwa angedauert? (Bitte in Minuten angeben z.B. 25, falls das Ereignis noch andauert, bitte gesamte Zeitdauer schätzen) | open |
| Alcohol consumption | Haben Sie in den letzten zwei Stunden Alkohol getrunken? | nein (0); ja (1) |
| Caffeine consumption | Haben Sie in den letzten zwei Stunden koffeinhaltige Getränke (z.B. Kaffee oder Energy Drinks) getrunken? | nein (0); ja (1) |
| Nicotine consumption | Haben Sie in den letzten zwei Stunden Zigaretten geraucht? | nein (0); ja (1) |
| Ambient noise, first diary of the day (8 am) | Waren Sie in den letzten zwei Stunden sehr lauten Umgebungsgeräuschen ausgesetzt? | nein (0); ja (1) |
| Ambient noise | Waren Sie seit der letzten Befragung sehr lauten Umgebungsgeräuschen ausgesetzt? | nein (0); ja (1) |
| Anticipated stress | Ich erwarte, dass die nächsten zwei Sunden stressig werden. | nein (0); ja (1) |
